# The Potential of Essential Oils from Active Packaging to Reduce Ethylene Biosynthesis in Plant Products. Part 2: Fruits (Blueberries and Blackberries)

**DOI:** 10.3390/plants12193418

**Published:** 2023-09-28

**Authors:** Antonio López-Gómez, Alejandra Navarro-Martínez, Alberto Garre, Asunción Iguaz, Ginés Benito Martínez-Hernández

**Affiliations:** 1 Food Safety and Refrigeration Engineering Group, Department of Agricultural Engineering, Universidad Politécnica de Cartagena, Paseo Alfonso XIII 48, 30203 Cartagena, Spain; antonio.lopez@upct.es (A.L.-G.); alejandra.navarro@upct.es (A.N.-M.); asun.iguaz@upct.es (A.I.); 2 Institute of Plant Biotechnology, Camouys Muralla del Mar (Universidad Politécnica de Cartagena), 30202 Cartagena, Spain; 3 Department of Agricultural Engineering, Universidad Politécnica de Cartagena, Paseo Alfonso XIII, 48, 30203 Cartegan, Spain; alberto.garre@upct.es

**Keywords:** cyclodextrin inclusion complex, plant essential oils, active packaging, ACC oxidase, ACC synthase

## Abstract

Plant essential oils (EOs) have an important ability to inhibit ethylene biosynthesis. Nevertheless, the effects of EOs on the key components of ethylene biosynthesis (l-aminocyclopropane-1-carboxylic (ACC) oxidase activity, ACC synthase activity, and ACC content) have not yet been thoroughly studied. Accordingly, this study focused on the effects of emitted EOs from active packaging (EO doses from 100 to 1000 mg m^−2^) on the key components of ethylene biosynthesis of blueberries and blackberries under several storage temperatures. Anise EO and lemon EO active packaging induced the greatest inhibitory effects (60–76%) on the ethylene production of blueberries and blackberries, respectively, even at high storage temperatures (22 °C). In terms of EO doses, active packaging with 1000 mg m^−2^ of anise EO or lemon EO led to the highest reduction of ethylene production, respectively. At 22 °C, the investigated EO active packing reduced the activities of ACC synthase and ACC oxidase up to 50%. In order to minimise ethylene biosynthesis in blueberries and blackberries when they are stored even under improper temperature scenarios at high temperatures, this EO active packaging is a natural and efficient technological solution.

## 1. Introduction

The disturbingly elevated rates of global food loss and waste demand prompt attention on a global scale. Food loss is defined by FAO [1] as occurring throughout the initial phases of the food supply chain, including production, postharvest storage, transportation, and processing. The end of the food supply chain, including retail and consumption, is where food waste occurs [1,2]. In particular, fruit- and vegetable-related retail and consumption phases make up 20–40% of the total amount of food lost and wasted [1]. As a result of these high rates, the United Nations issued an ambitious challenge in 2015 to reduce the amount of food wasted globally per person by half by 2030 [3]. The main factors contributing to the significant amount of fruit and vegetable waste are the ripening and senescence processes, which cause products to lose quality over their postharvest lives [2,4]. A key factor in the postharvest quality decline of horticultural commodities is ethylene, which is known as the ripening hormone of plant goods [5].

The enzyme ACC synthase (ACS) converts S-adenosyl-L-methionine into l-aminocyclopropane-1-carboxylic acid (ACC), which is the first step in the ethylene biosynthesis pathway. The ACC N-Malonyl transferase can then convert ACC to 1-(malonylamino)cyclopropane-1-carboxylic acid or the final product, ethylene, via the ACC oxidase (ACO) [6,7]. Additionally, recent investigations have revealed evidence of additional ACC-derivative products (different from ethylene or 1-(malonylamino)cyclopropane-1-carboxylic acid), including -glutamyl-ACC, jasmonyl-ACC, and bacterial metabolization products (-ketobutyrate and ammonium) [8]. However, ACS and ACO are the two key enzymes for ethylene production in plant products, according to substantial evidence [8]. It has been reported that ACO is the rate-limiting phase, such as during the post-climacteric ripening of tomato fruit, although it is widely acknowledged that ACS is the rate-limiting stage of ethylene production in plants [8].

Grouping different fruits and vegetables together in one location for postharvest storage and distribution is a very popular practice that helps keep costs down when there are fewer products being produced. However, it must be carried out carefully because mixing components with various ethylene production rates could have a negative impact on the products’ quality. Ethylene production of blueberries is highly dependent on the cultivars and other preharvest factors ranging published data from 5 to 50 pmol kg^−^^1^ s^−^^1^ [9,10,11]. Contrarily, higher ethylene production rates have been reported for blackberries [12]. Several solutions have been put forth to lessen the impacts of ethylene and can be divided into two categories: (i) lowering the amount of ethylene produced by the plant product, or (ii) removing the produced ethylene from the atmosphere around the plant product [5,13]. However, the majority of these methods are expensive and/or rely on chemical goods, which the actual consumer, who prefers more natural products devoid of chemical synthesis additives, may reject [14]. It is interesting to note that plant essential oils (EOs) have a strong potential to lower the synthesis of ethylene in plant-based products [15,16,17,18].

Plant EOs are natural extracts that have well-known antibacterial properties and are highly prized by customers as natural additives. Strong in vitro antibacterial activity of EOs and EO compounds, which has been widely explored and documented in the literature [19], is effective against a wide variety of spoilage microorganisms and pathogens. Additionally, EO treatments have been used to preserve quality aspects like colour, firmness, and others in fruit and vegetables after harvest [20,21]. The European Union has approved most of the EOs and their main EO constituents as food additives. The European Union specifically classifies EOs and EO compounds as “natural flavouring substances” and “flavouring preparations”, respectively [22,23,24]. High EO doses, however, require special caution because the customer may detect the characteristic EO off-flavours or aromas in the treated product. However, encapsulation of EOs (e.g., using cyclodextrins) may avoid such sensory disadvantages due to the minor but effective burst-avoiding effect, EO concentrations released from the EO encapsulation system [25].

Active packaging involves adding active compounds (antimicrobial, antioxidant, or other preservative properties) to the packaging material and subsequent controlled release during the product’s shelf life [26]. Our research studied and validated at the industrial level active paper/cardboard packaging with encapsulated EOs (within cyclodextrin) in recent years, successfully extending the shelf life of fresh fruit and vegetables [17]. The EO release kinetics of this active paper/cardboard packaging technology at various EO doses (100–1000 mg m^−^^2^) and relative humidity scenarios (50–60% and 90–95%), which are typical during the retail and distribution of fruit and vegetables, have been fully characterised [25]. This EO-active paper/cardboard packaging technology also decreased the formation of ethylene in fruits and vegetables [15,18]. Since the regular price of a cardboard box is only increased by 3–5%, using this EO active packaging technology as an option to minimise ethylene output is economically justified. However, a thorough examination of the impact of the released EOs (from active packaging) on the ethylene biosynthesis pathway, including ACC, ACO, ACC, and associated ethylene production, has not yet been conducted.

This research aims to investigate the impact of EOs, previously encapsulated within β-cyclodextrin, released from an active cardboard packaging on the ethylene biosynthesis pathway key components (ACC, ACO, ACC and associated ethylene production) of blueberries and blackberries at various temperatures. Hence, several active packaging with various EOs (lemongrass EO, anise EO, fennel EO, lemon EO, citral, and geraniol) and doses (100–1000 mg m^−^^2^) were tested.

## 2. Results and Discussion

### 2.1. ACS Activity

The role of ACC in the ethylene biosynthesis pathway is well characterized since its discovery in 1979 [6]. Nevertheless, there are still many questions concerning the ethylene biosynthesis pathway key enzymes: ACS and ACO [8]. The ACS can be considered a key enzyme of the ethylene biosynthesis pathway, which converts SAM into ACC [7,27]. It is well accepted that ACS is the rate-limiting step of ethylene biosynthesis in plants, although there are examples where ACO role is high in fruits like blueberries and blackberries [10,11,12,28]. In particular, Wang et al. [28] pointed out that an increase in ethylene evolution of blueberries was associated with increases in transcript abundance of its biosynthesis genes related to ACS and ACO activities: ACS1 and ACO2, respectively. Hence, storage at 2 °C increased the ACS-related gene expression by 1.7-fold after 4 days (5.6-fold after 8 days) in blueberries [11], although the ACS activity remained unchanged (*p* > 0.05) at lower storage temperatures (0 °C) up to 60 days [10].

**Figure 1:**
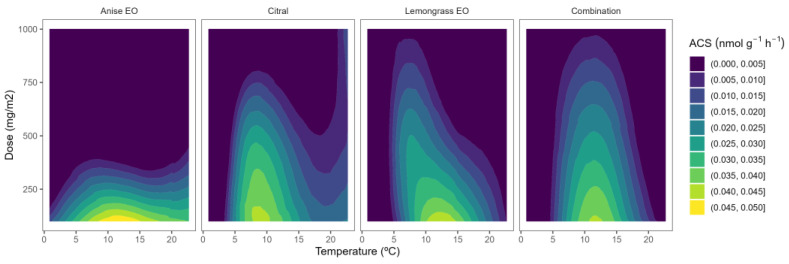
Surface responses for ACS activity of blueberries packaged with active packaging including different essential oils (citral, lemongrass, anise and their combination) under different temperatures.

The ACS activity of blueberries ranged from 0.16 to 0.20 nmol g^−^^1^ h^−^^1^ without differences (*p* > 0.05) among temperatures. Similarly, Wang et al. [10] did not report high differences in the ACS activity of blueberries (cv. Lanfeng) stored either at 2 or 20 °C, which ranged from 3.02 to 3.70 nmol kg^−^^1^ s^−^^1^. Higher temperatures than 15 °C showed lower ACS activity, although those changes were not significant (*p* > 0.05). As expected, temperature reduction (below 10–15 °C) led to lower ACS activity in blueberries as observed in Figure 1 from the yellow-to-blue colours. Nevertheless, the opposite behaviour was observed at higher temperatures (>10–15 °C), which may be explained by a previous high SAM consumption by ACS in the previous hours of sampling (sampling times were made after 5–30 h of ethylene accumulation as explained in Section 3.3).

The ACS activity in blueberries was reduced using EOs active packaging (Figure 1). Hence, the observed yellow-to-blue turning from low to high EOs doses was observed for all the studied EOs. Among them, a strong ACS activity inhibition–EOs dose correlation was observed for the anise EO active packaging. This high correlation was even stronger at higher temperatures with inhibition percentages of ACS activity of 28/31/32, 24/31/42, 20/36/53, and 36/34/53% for 100/500/1000 mg m^−^^2^ doses at 2, 8, 15, and 22 °C, respectively. As observed, anise EO active packaging led to the inhibition of ACS activity of 40–50% at the highest studied EO dose (1000 mg m^−^^2^). The effect of EOs on the ACS activity of blueberries has not been previously studied to the best of our knowledge.

In blackberries, the ACS activity ranged from 0.40 to 0.29–0.33 nmol kg^−^^1^ s^−^^1^ at 22 and 2–15 °C, respectively (data tables included as Table S1). There are no published ACS activity data related to blackberries to the best of our knowledge. Similar to blueberries, the temperature reduction seemed to reduce the ACS activity, although this trend was not very intense compared with other vegetables [15]. No good data fitting was observed for the ACS data of blackberries, being presented as tabulated data in Table S1. The active packaging induced little inhibition (<20%) of the ACS activity in blackberries at temperatures lower than 22 °C (Table S1). Nevertheless, EO active packaging reduced ACS activity by 30–50% at 22 °C, without a clear ACS activity inhibition–EO dose correlation. In that sense, low EO doses induced the same effects as higher EO doses on the ACS activity inhibition, inducing lemon EO active packaging at 100 mg m^−^^2^ with a reduction of the ACS activity of 49% at 22 °C.

### 2.2. ACC Content

A significant development in our understanding of the process by which ethylene is produced in plants was the discovery of ACC as its precursor, which served as a major building block for other subsequent ethylene biology discoveries [8]. The initial ACC contents of control blueberries ranged from 0.012 to 0.048 nmol g^−^^1^, which is in agreement with previous literature for blueberries at the ripe stage [28]. Interestingly, the ethylene biosynthesis metabolism during the ripening of blueberries has been shown to greatly vary among different fruit varieties [28]. In particular, Wang et al. [28] found high variations among rabbiteye blueberries “Premier” and “Powderblue” cultivars with ACC values of 1 and <0.05 nmol g^−^^1^, respectively, in the pink ripening stage.

**Figure 2:**
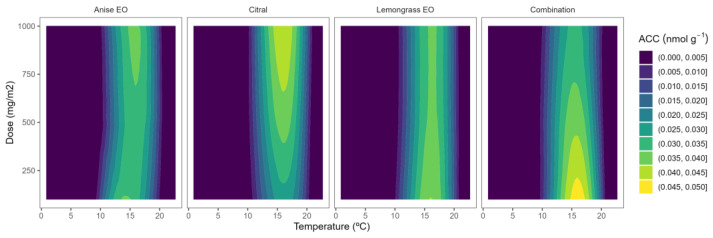
Surface responses for ACC contents of blueberries packaged with active packaging with different doses of inclusion complexes of different essential oils (citral, lemongrass, anise, and their combination) under different temperatures.

With regard to temperature, greater ACC values were observed at higher temperatures up to 15 °C (Figure 2). Similarly to ACS, a different behaviour was observed in temperatures higher than 15 °C, which may be explained by a previous high ACC-to-ethylene conversion by ACO in the previous hours of sampling. The ACC contents (Figure 2) are strongly related to ACS activity data (Figure 1).

With regard to the EO dose effect, a different ACC content behaviour was observed compared with ACS activity. In particular, no remarkable ACC content variations (low-curved lines) were observed (at the same temperature) when increasing the EO dose (Figure 2). In that sense, low EO doses achieved the same effects as higher doses (with the consequent EO cost reduction) to reduce the ACC formation, the precursor of ethylene [15]. The inhibitory effect of EOs on the ACC content has been previously reported in apples and flat peaches [15,17,18]. The effect of EOs on the ACC content of blueberries has not been previously studied to the best of our knowledge.

**Figure 3:**
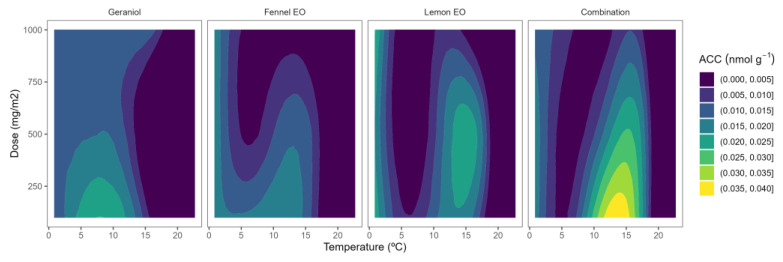
Surface responses for ACC content of blackberries packaged with active packaging including different essential oils (geraniol, fennel, lemon, and their combination) doses under different temperatures.

The initial ACC contents of control blackberries ranged from 0.022 to 0.076 nmol g^−^^1^. These values are higher than previously published data for ripened blackberries (0.5 nmol g^−^^1^) [12], which may be due to variety-dependent and preharvest factors (growing area, harvest time, etc.), as observed in other fruits [29]. As previously noted, a high SAM-to-ACC conversion may be observed in the first 5–30 h, with lower values at high temperatures (>15 °C) (Figure 3) and consequent ACC displacement to ethylene production. In general, and similarly to blueberries, a clear EO dose effect was not observed for blackberries, showing that all EOs have similar behaviour.

### 2.3. ACO Activity

**Figure 4:**
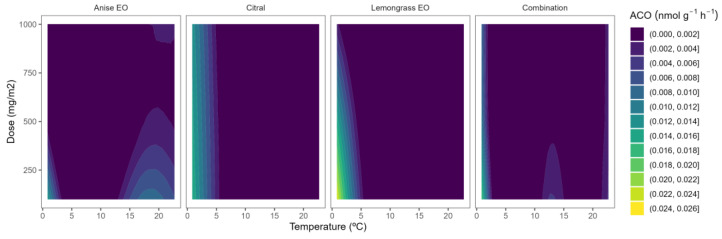
Surface responses for ACO activity of blueberries packaged with active packaging with different doses of inclusion complexes of different essential oils (citral, lemongrass, anise, and their combination) under different temperatures.

ACO is the other key enzyme, in addition to ACS, of the ethylene biosynthesis pathway. Wang et al. [28] reported that ACO is inducible by ethylene in blueberries and that a potential autocatalytic response is functional at the level of ACO. Indeed, postharvest storage of blueberries at 0–2 °C led to 3- and 5.6-fold ACO activity increments after 8 and 15 days, respectively [10,11]. The ACO activity of control blueberries was 0.020–0.026 nmol g^−^^1^ h^−^^1^. In general, no remarkable effects of the used EO or EO doses were observed on the ACO activity with low-curved lines and predominance of bluer colours in Figure 4.

**Figure 5:**
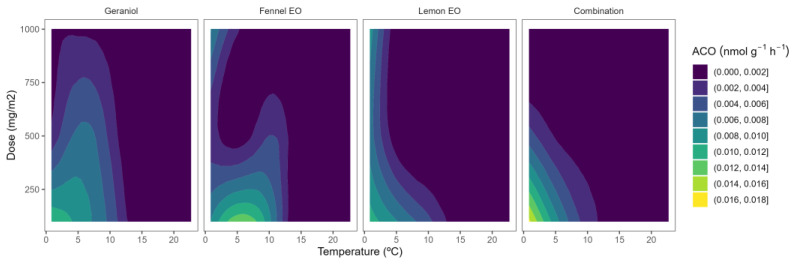
Surface responses for ACO activity of blackberries packaged with active packaging including different essential oils (geraniol, fennel, lemon, and their combination) doses under different temperatures.

In blackberries, as for other fruits, it has been previously reported that ACO appears to control ACC levels throughout the last phases of ripening [12]. According to Burdon and Sexton [30], the limiting step in the generation of ethylene in blackberries is the activity of the enzyme ACO. Perkins-Veazie et al. [12] reported that ethylene production of blackberries (‘Navaho’ cv.) increased somewhat before ACO activity and ACC contents increased. The ACO activity of our samples ranged from 0.012 to 0.030 nmol g^−^^1^ h^−^^1^. Lower ACO activity levels have been previously reported in other blackberry cultivars [12], which may be due to the influence of the ripening stage or other preharvest factors as previously explained. As observed in Figure 5, yellow-blue colour (high ACO activity) varied to bluer colour tones (low ACO activity) as the temperature increased. This behaviour corroborates a rapid ACC-to-ethylene conversion before the sampling time for blackberry samples (1 h; see Section 3.3), which was reduced at lower temperatures, remaining as unconverted ACC to still allow some ACO activity. The EOs’ capacity to reduce the ACO activity has not been widely studied in the literature, having only studied in apples and flat peaches [15,17,18]. The effects of EOs on the ACO activity of blueberries and blackberries have not been previously studied.

Among EOs, lemon active packaging showed a clearer EO dose effect, reducing the ACO activity as the lemon EO content was increased (Figure 5). In particular, the lowest level of ACO activity (≈0.01 nmol g^−^^1^ h^−^^1^) was observed at the highest lemon EO dose of 1000 mg m^−^^2^, meaning that the EO dose influenced the ACO activity higher as the temperature increased.

### 2.4. Ethylene Production

The ethylene production of blueberries was 0.16, 0.7–0.8, and 2.6 nmol kg^−^^1^ s^−^^1^ at 2, 8–15 and 22 °C, respectively. Other blueberry varieties showed lower ethylene production rates [9], which may be due, as previously explained, to different ripening stages and several preharvest factors [28]. The ethylene production evolution, together with changes in the respiration rate, are congruent with climacteric fruit ripening among numerous blueberry genotypes [28]. In particular, increased ethylene evolution during blueberry fruit ripening is linked to changed transcript abundance of genes relevant to ethylene production, perception, and signalling, suggesting functional ethylene signalling during blueberry ripening [28]. Therefore, ethylene is likely to influence the blueberry fruit ripening syndrome.

**Figure 6:**
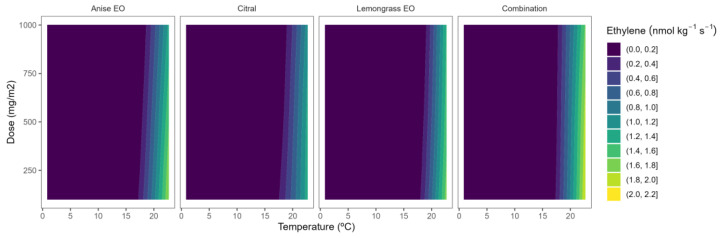
Surface responses for ethylene production of blueberries packaged with active packaging with different doses of inclusion complexes of different essential oils (citral, lemongrass, anise, and their combination) under different temperatures.

**Figure 7:**
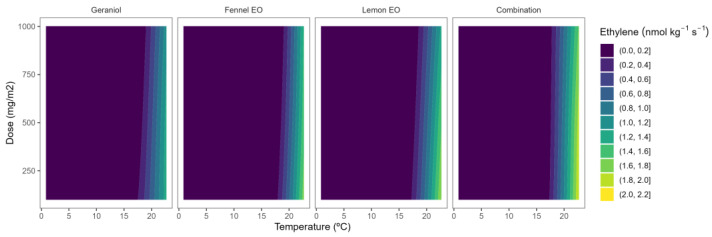
Surface responses for ethylene production of blackberries packaged with active packaging including different essential oils (geraniol, fennel, lemon, and their combination) doses under different temperatures.

A clear ethylene production–temperature correlation was observed with greater ethylene production (yellow-green colour tones) at higher temperatures, compared to lower ones, in both blueberry and blackberry samples (Figure 6 and Figure 7). In particular, blackberry ethylene production at 22 °C was reduced by 3–3.5 at 8–15 °C, and up to 16-fold at 2 °C. Nevertheless, lesser reductions (1.1–1.8-fold) were observed for blackberries when the temperature was decreased. Reduction of storage temperature is a well-known postharvest procedure to reduce the postharvest metabolism, especially ethylene production, of fruit and vegetables [5]. Alternatively, we propose the use of EOs to reduce the ethylene production of horticultural products to reduce energy costs by increasing the setpoint temperature during the storage of these products [18].

Among EOs, fennel EO induced the highest ethylene production inhibition rates (40–75%) in blueberries, showing the surface response with slightly higher curved patterns compared with the rest of EOs (Figure 6). In particular for fennel EO, no clear EO dose effect was observed at the lowest storage temperature with inhibition rates of ethylene production of 60–76%. However, higher temperatures were needed to increase the EO dose to maintain high ethylene inhibition rates (>40%), with inhibition values of 40–55% for 1000 mg m^−^^2^ doses at 15–22 °C.

In blackberries, the highly varied ACC contents and ACO activity found in the receptacle and drupelet tissues suggest that ethylene generation and/or sensitivity are spatially and temporally separated. Ripening starts in the fruit receptacle, as evidenced by the fact that it softened more quickly and had lower hardness values than the drupelets. Although ripening in blackberries is probably independent of ethylene, ethylene may regulate berry detachment from pedicels, thus controlling the fruit harvest time [12]. The ethylene production of blackberries was 835.0 nmol kg^−^^1^ s^−^^1^ at 20 °C. As observed in Figure 7, the high ethylene production at 22 °C was reduced as the temperature was decreased, turning from yellow to blue colours.

Among EOs, lemon EO induced the highest inhibition of ethylene production in blackberries with reductions that ranged from 30–60%. In addition, lemon EO showed the highest ethylene production inhibition–EO dose correlation, as observed from the higher curvature of lines (Figure 7). In particular, as the dose increased, the ethylene production decreased, inducing the 1000 mg m^−^^2^ dose 50–60% reductions in the ethylene production, without remarkable differences in the temperatures. In that sense, lemon EO active packaging at 100 mg m^−^^2^ had almost the same high capacity (50–60%) to reduce the ethylene production in blackberries independently if they are stored under refrigeration or room temperature.

## 3. Materials and Methods

### 3.1. Materials

Plant EOs (lemongrass, anise, fennel and lemon EOs) were acquired from Esencias Martínez Lozano S.A. (Caravaca de la Cruz, Spain). EO composition analyses are included in Table S2. Citral (99.5% purity) and geraniol (99.5% purity) were acquired from Merck (Dusseldorf, Germany). β-cyclodextrin, hereinafter referred to as βCD, was provided by Roquette (Kleptose^®^10; Lestrem, France). Water-base lacquer UKAPHOB HR 530 (ammonia-free anionic copolymer; pH 8–10; viscosity max. 100 mPa s at 20 °C; with 30% total solids concentration), which is authorized for food contact surfaces, was acquired from Schill+Seilacher GmbH (Böblingen, Germany). This is a common lacquer type used for paper/cardboard packaging of fruit and vegetables with the following advantages: (i) it is easily dissolved in water to reach the appropriate density to ensure homogeneous spraying on the cardboard surface; and (ii) when dried, it improves impermeabilization of the paper/cardboard surface against the high humidity levels maintained (to reduce water loss of plant products) in the cold rooms of horticultural facilities. Recycled kraft paper sheets (50 g m^−^^2^) were provided by Bioencapsulation and iPackaging S.L. (Fuente Álamo, Murcia, Spain).

Blueberries (*Vaccinium corymbosum*) were acquired in a local supermarket (Mercadona; Cartagena, Spain) in January 2023. According to the traceability data consulted by the producer, blueberries were grown in the southwest Spain area (Huelva) and harvested manually. Blackberries (*Rubus fruticosus* var. Equa) were acquired from the company, Plus Berries (Aljaraque, Spain), in March 2023. Blackberries were transported from the company in a refrigerated (2 °C) vehicle for 7 h (650 km) to the Universidad Politécnica de Cartagena.

### 3.2. Encapsulation of Essential Oils and Active Packaging Preparation

The EOs and EO components used for the active packaging in blueberries (citral, lemongrass EO, anise EO and their combination in a ratio of 3:1:1 weight(*w*):*w*:*w* corresponding the major proportion to the EO component) and blackberries (geraniol, fennel EO, lemon EO and their combination in a 3:1:1 *w*:*w*:*w* ratio) were selected based on their high capacity to inhibit the ethylene production in these vegetables based on preliminary experiments with approximately 50 different EOs (and different combinations of them) (data not shown).

The EO−βCD inclusion complex was prepared using the kneading method [31]. Briefly, 1 g of EO was mixed with 7.6 g of βCD (following a 1:1 EO:βCD molar ratio) in a mortar with 3 mL of ethanol, kneaded for 45 min and finally maintained in a vacuum desiccator at room temperature for least 72 h (it reduces the surface EO that is not trapped in the βCD cavity). This EO−βCD inclusion complex prepared using the same methodology and equipment has been characterized (first part of this article, https://doi.org/10.3390/plants12193404).

The EO−βCD inclusion complex was dissolved (at different concentrations as subsequently shown) in diluted lacquer before spraying on the kraft paper. The lacquer was previously diluted to a final solid concentration of 8.5% to compensate for the addition of the EO−βCD inclusion complex as lacquers with solid content ≥ 30% may be difficult to spray on the paper or cardboard surface.

The active packaging was prepared to obtain load levels of the EO−βCD inclusion complex of 100–1000 mg m^−^^2^, which would be equivalent (based on the previous EOs:βCD molar ratio of 1:7.6) to entrapped 11.6–116.3 mg of EOs per m^2^ of paper. The selected load range of the EO−βCD inclusion complex was selected between the minimum dose to observe the benefits on the product quality during storage and the maximum dose without transferring EO-related off-flavors to the product as previously reported [25]. Active packaging material was prepared one day before the experiments.

### 3.3. Effect of Active Packaging on the Ethylene Biosynthesis System of Vegetables

Blueberries (≈130 g) and blackberries (≈110 g) were packaged in rectangular plastic baskets (120 × 110 × 45 mm; 1 L of capacity). A rectangle (120 × 110 mm) of active packaging was placed in the bottom of the basket before filing with the blueberries and blackberries. Subsequently, trays were thermal-sealed with an automatic packaging machine (Efaman; Efabind, Murcia, Spain) with a Cryovac^®^ EOP616B film (39 µm thickness; Cryovac, Fuenlabrada, Spain) with synthetic air. The gas/water transmission rates of this film were O_2_, 7.0 cm^3^ m^−^^2^ day^−^^1^ atm^−^^1^; CO_2_, 25.0 cm^3^ m^−^^2^ day^−^^1^ atm^−^^1^; N_2_, 0.5 cm^3^ m^−^^2^ day^−^^1^ atm^−^^1^; water, 10.0 g m^−^^2^ day^−^^1^.

Sampling for analyses (ethylene production, ACC content, and ACO and ACS activity) of blueberries was performed after 48, 30, 24, and 5 h of ethylene accumulation at 2, 8, 15, and 22 °C, respectively. For blackberries, sampling was made after 1–2 h for all storage temperatures (2, 8, 15 and 22 °C). The selected accumulation times were based on preliminary tests to achieve measurable ethylene concentrations within packages but avoiding CO_2_ and O_2_ consumption concentrations higher/lower than 5/15% (measured with a O_2_/CO_2_ portable meter; Checkpoint O_2_/CO_2_ model, PBI Dansensor, Barcelona, Spain), respectively, to avoid alteration of the normal product metabolism.

### 3.4. Ethylene Production

The ethylene production was analysed according to previous literature [16]. Briefly, 1 mL gas was taken from the headspace of the containers using a gas-tight syringe and then injected into a gas chromatograph (GC; Clarus 500 GC; Perkin Elmer Inc., Shelton, CT, USA) for the ethylene determination. Two measurements (technical replicates) were made for each basket.

The GC included a GC column (stainless steel column packed with Porapak Q 1/8″, 80/100 mesh size; Teknokroma; Barcelona, Spain), and the analysis conditions were oven, injector and flame ionization detector temperatures of 80, 120, and 250 °C, respectively, with synthetic air and H_2_ as gas carriers at 350 and 35 mL min^−1^, respectively. Ethylene content was quantified using an ethylene standard of 10 ppm (gas molar fraction volume) (Praxair; Molina de Segura, Spain). The results were reported as nmol g^−^^1^ h^−^^1^.

After collecting samples for the ethylene analyses, the baskets were opened and samples were frozen using liquid nitrogen. Then, samples were kept at −80 °C until ACC content and enzymatic analyses (ACO and ACS) were performed.

### 3.5. 1-Aminocyclopropane-1-Carboxylic Acid (ACC) Content

The original approach developed by Lizada and Yang [32] (reviewed by Bulens et al. [33]) was used to determine the ACC content. For the ACC extraction, 4 g of ground (IKA A11 Basic; mill with liquid N2; Königswinter, Germany) frozen tissue was homogenised (IKA T-18 digital ULTRA-TURRAX^®^; Königswinter, Germany) with 4% salicylic acid solution (in distilled water), followed by vortexing and being placed on ice for 30 min while being stirred. Samples were then centrifuged at 3090× *g* for 10 min at 4 °C. In order to measure the ACC content, 1.4 mL of ACC extract and 0.4 mL of 10 mM HgCl_2_ were combined in a 20 mL-glass GC vial before being immediately sealed with an encapsulable septum. After adding 0.2 mL of NaOCl (5% volume (*v*):*v*):NaOH (6 M) mix (2:1 *v:v*) using a syringe, the reaction began. This was followed by an incubation period of 4 min on ice. Ultimately, 1 mL of the GC vial headspace was injected in the GC and the generated ethylene was analysed (as indicated in Section 3.4). The results were reported as nmol g^−^^1^.

### 3.6. ACC Oxidase (ACO) Activity

The ACO activity was analysed as described in the literature [33,34]. For the ACO extraction, 0.5 g of ground frozen sample, plus 50 mg of polyvinylpolypyrrolidone, were added to 1 mL of MOPS (3-morpholinopropane-1-sulfonic acid) buffer (400 mM, pH 7.2), which contained 10% glycerol (*w*:*v*) and 30 mM sodium ascorbate. Then, it was shaken for 15 min at 4 °C. Samples were then centrifuged at 22,000× *g* for 30 min at 4 °C, and the supernatant was used as the ACO extract. The ACO reaction was started by mixing 0.4 mL of the ACO extract with 3.6 mL of ACO reaction buffer (50 mM MOPS buffer comprising 10% glycerol, 1 mM ACC, 10 mM sodium ascorbate, 50 µM iron sulphate, 10 mM sodium bicarbonate and 1 mM dithiothreitol; pH 7.2) in a 20 mL glass GC vial, which was then rapidly closed with an encapsulable septum. The reaction lasted for 1 h at 30 °C in a water bath. Ultimately, 1 mL of the GC vial headspace was injected in the GC, and the generated ethylene was analysed (as indicated in Section 3.4). The results were reported as nmol g^−^^1^ h^−^^1^.

### 3.7. ACC Synthase (ACS) Activity

The ACS activity was analysed as previously described [33,34]. For the ACS extraction, 3 g of ground frozen sample, plus 15 mg of polyvinylpolypyrrolidone, were added to 3 mL of the extraction buffer, which consisted of 200 mM tricine (pH 8.5) containing 4 μM pyridoxal-L-phosphate and 10 mM dithiothreitol. It was then stirred for 15 min at 4 °C. Samples were then centrifuged at 22,000× *g* for 30 min at 4 °C, and the supernatant was used as the ACS extract. The ACS extract was purified using solid-phase extraction columns (Sephadex G-25 desalting column GE17-0851-01; Sigma-Aldrich, Berlin, Germany). For the reaction, 1.5 mL of the purified ACS extract was added to 150 µL of the ACS reaction buffer (tricine buffer 200 mM, pH 8.0) and 150 µL SAM chloride. The reaction continued for 2 h at 25 °C in a water bath. Finally, 200 µL of the 100 mM HgCl_2_ solution was added to stop the reaction. Subsequently, 950 mL of the reacted extract was mixed with 850 mL of distilled water in a 20 mL glass GC vial, which was then quickly sealed with an encapsulable septum. Then, 0.2 mL of the NaOH-NaOCl solution (see Section 3.5) was added through the septum and incubated for 4 min on ice. Ultimately, 1 mL of the GC vial headspace was injected in the GC and the generated ethylene was analysed (as indicated in Section 3.4). The results were reported as nmol g^−^^1^ h^−^^1^.

### 3.8. Data Analysis and Mathematical Modelling

Smoothing splines (b-splines) were used to describe how both storage temperature and the concentration of antimicrobial compounds affect the concentration of the measured compounds (ACC, ACO, MACC, ACS and ethylene). Smoothing splines were selected instead of more classical methods (e.g., polynomials) due to their better ability to describe complex non-linear relationships.

The models were fitted using Bayesian regression [35] to better account for the experimental error. The models were implemented in R version 4.2.3 [36] using the *brms* package [37]. Specifically, we used the “t2” implementation from the *mgcv* package [38] using the storage temperature and dose of antimicrobial as predictors and the concentration of the compound as the output variable. The model included a random effect [39] to account for the type of antimicrobial (or combination). An independent model was defined for each compound (ACC, ACO, MACC, ACCS, or ethylene).

Models were fitted using the *brms* package for R, which interfaces the No-U-Turn sampler from Stan [40]. Model convergence was evaluated by checking the trace plots of the Markov Chains and ensuring that the R-hat index was lower than 1.01 for every parameter estimate, as is often recommended for this modelling approach [35]. The parameter estimates of the fitted models are included in Table S3, and were represented as contour plots for analysis using *ggplot2* [41].

## 4. Conclusions

The effects of the released essential oils (EOs) from active packaging were studied on the key systems of the ethylene biosynthesis pathway (ACS, ACC, and ACO) in blueberries and blackberries. The greatest inhibitory effects on ethylene biosynthesis were observed for anise EO and lemon EO active packaging for blueberries and blackberries, respectively. Such inhibition biosynthesis was higher as the EO dose of the active packaging was increased for both fruits, with the maximum inhibition rates observed when using 1000 mg m^−^^2^. In particular, the active packaging reduced the activities of key enzymes, ACS and ACO, up to 50%, even at an unsuitable storage temperature of 22 °C. Therefore, storage of berry fruits like blueberries and blackberries using anise EO or lemon EO active packaging, respectively, may considerably lessen the negative impacts of ethylene on the postharvest quality of these delicate products. Future research may deepen into the genetic factors influencing the reported inhibition of the key enzymes ACS and ACO from the ethylene biosynthesis pathway.

## Data Availability

The data presented in this study are available on request from the corresponding author.

## Supplementary Materials

The following supporting information can be downloaded at https://www.mdpi.com/article/10.3390/plants12193418/s1, Table S1: ACS activity of blackberries; Table S2: Composition of the used essential oils; Table S3: Statistical analysis files.
