# Supplementary material for: The Potential of Essential Oils from Active Packaging to Reduce Ethylene Biosynthesis in Plant Products. Part 2: Fruits (Blueberries and Blackberries)
Source: Plants (Basel). 2023 Sep 28;12(19):3418. doi: 10.3390/plants12193418 (PMC10574652; doi:10.3390/plants12193418)
Supplement: Supplementary file 1 [file plants-12-03418-s001.zip › Table S2.pdf]

Lemongrass essential oil

Component	Content (%)
limonene	0.5 - 3
6-methyl-5-hepten-2-one	0.1 - 2
β-caryophyllene	0.2 - 3.2
Neral	25 - 35
Geranial	25 - 47
Acetate of geranyle	0.6 - 6
Geraniol	1.5 - 8

Grapefruit essential oil

Component	Content (%)
Trans-anetol	97 - 94
g-himalacheno	1 – 5
Estragol	0.5 - 3
Anisaldehído	0.1 – 1.4
Cis-Anetol	0.1 – 0.4

Fennel essential oil

Component	Content (%)
α-pineno	1 - 8
β-pineno	0 - 1
α -felandreno	0.2 - 5
Limoneno	1 - 8
Fenchona	8 - 20
Estragol	2 - 6
Cis-anetol	0 - 0.5
Trans-anetol	60 - 80
Anisaldehyde	0 - 2

Lemon essential oil

Component	Content (%)
α-pinene	1.5 - 3
β-pinene	10 - 16
Sabinene	1.5 - 3
Limonene	60 - 70
p-cymene	0 - 0.4
γ-terpinene	8 - 12
Neral	0.4 - 1
α -terpineol	0.1-0.3
Geranial	0-1
Acetate of neryle	0.6 - 2
